# Nitrous Oxide-Induced Vitamin B12 Deficiency Resulting in Myelopathy

**DOI:** 10.7759/cureus.9088

**Published:** 2020-07-09

**Authors:** Victoria Campdesuner, Yeshanew Teklie, Talal Alkayali, Derek Pierce, Justin George

**Affiliations:** 1 Internal Medicine, Sarasota Memorial Hospital, Florida State University College of Medicine, Sarasota, USA

**Keywords:** nitrous oxide, vitamin b12, vitamin b12 deficiency

## Abstract

Nitrous oxide, primarily used in the medical field and in the food industry, can cause symptoms of euphoria and analgesia. Recreational use of nitrous oxide is rising, as are reports of its adverse effects, including neurologic complaints secondary to an evoked vitamin B12 deficiency. We outline a case of a patient presenting with several neurologic symptoms and found to have myelopathy secondary to vitamin B12 deficiency in the presence of prolonged recreational nitrous oxide use.

## Introduction

Discovered in the late 1700s, nitrous oxide, often referred to as “whippets” or “laughing gas”, is a colorless gas known for its anesthetic and analgesic use in the medical field [1-2]. Due to its bacteriostatic effect, it is used in the food industry as a foaming agent and spray propellant in aerosol canisters, such as those containing whipped cream [1-3]. Because of its ability to cause euphoria and its widespread availability, recreational nitrous oxide use is escalating. Metal canisters filled with nitrous oxide are sold online and at supermarkets and consumers often inhale the canisters’ contents to achieve intoxication [3]. 

Though recreational use of nitrous oxide is reported to be safe compared to that of other illicit substances, its use has been associated with several adverse effects, including confusion, hallucinations, and falls [3]. Fatalities associated with nitrous oxide use have been attributed to accidental asphyxiation due to oxygen displacement, often resulting in arrhythmias and seizures [3]. One of the most clinically significant adverse effects of nitrous oxide use is its association with vitamin B12 deficiency and resulting paresthesia, myelopathy, and subacute combined degeneration of the spinal cord [1]. 

## Case presentation

A 42-year-old female with a past medical history significant for restless leg syndrome, anxiety, depression, and hepatitis C presented to the ED with multiple neurologic complaints including headache, neck pain, speech difficulty, and ataxia for six weeks. Headache was described as constant, throbbing, and diffuse encompassing the sinuses, frontal, and occipital regions. There was no associated photophobia or phonophobia. Her neck pain was primarily para-cervical and radiated just posterior to her ears bilaterally. Speech difficulties included an inability to finish sentences, though word finding difficulty was denied. The patient reported difficulty with ambulation, primarily imbalance associated with numbness. She described feeling as if her legs gave way beneath her, resulting in multiple falls and injuries to her knees. She denied any fevers, chills, head trauma, weight loss, or blurred vision. She denied smoking, alcohol consumption, or illicit drug use initially. However, a few days following admission, the patient admitted to using nitrous oxide recreationally for over two years.

On examination, the patient was alert and oriented, in no acute distress. Speech was fluent and nondysarthric. Naming, repetition, attention, and insight were intact. Cranial nerves II-XII were intact. All four extremities demonstrated good muscle bulk and tone with 5/5 strength. No pronator drift, tremors, or abnormal movements noted. Light touch sensation was intact in all extremities, though there was diminished vibratory sensation in the lower extremities. There was no dysmetria on finger-to-nose and no ataxia on heel-to-shin. The patient was unstable upon standing and was noted to have an ataxic gait. Otherwise, physical exam was unremarkable.

**Figure 1:**
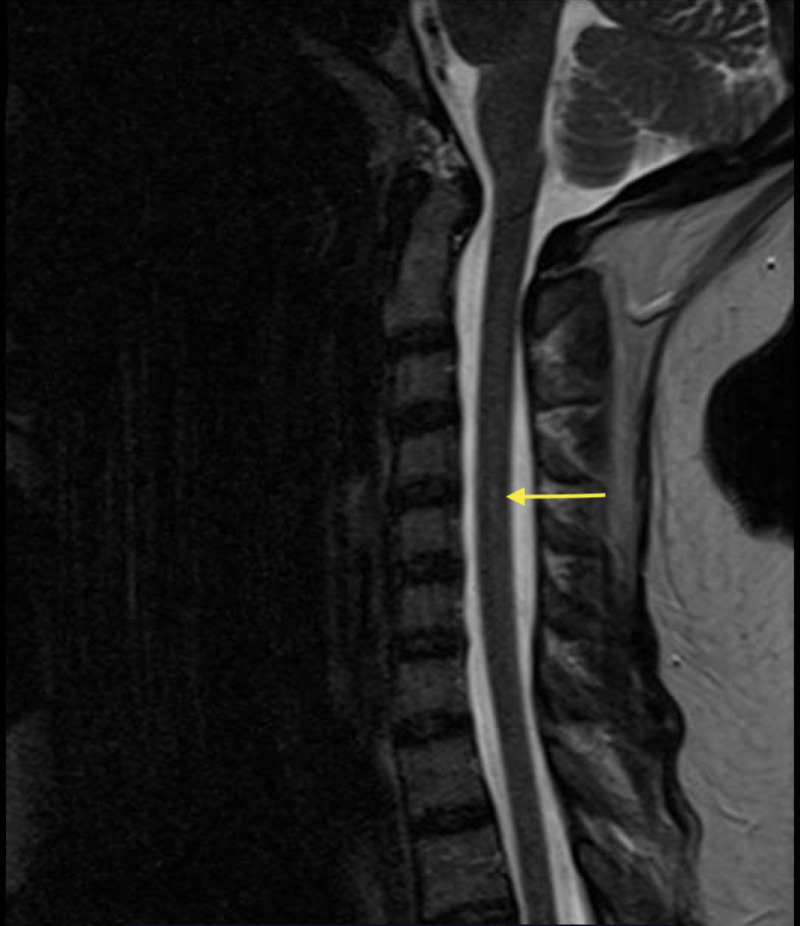
MRI of the cervical spine showing focal central cord T2 signal hyperintensity at C4-C5.

Laboratory studies revealed a normal complete blood count with a hemoglobin of 14.6 (12.3-15.3 g/dL) and mean corpuscular volume (MCV) of 93.8 (80-100 fL). Electrolytes, renal and liver function tests were within normal limits. Serum syphilis antibody was nonreactive. MRI of the brain without contrast showed a large right mastoid effusion, consistent with mastoiditis, for which the patient received treatment with IV vancomycin and cefepime. Magnetic resonance angiography (MRA) of the brain showed no evidence of large vessel occlusion. Lumbar puncture was performed with normal cerebrospinal fluid (CSF) studies, including white blood cell count, glucose, protein, and opening pressure. Polymorphonuclear cells were elevated at 75%, however, there was no evidence of an infectious process with negative CSF acid-fast bacilli culture, bacterial culture, CSF venereal disease research laboratory (VDRL) test, viral meningitis/encephalitis panel, cryptococcal antigen, Herpes Simplex Virus 1, 2 testing via CSF polymerase chain reaction (PCR), and Varicella zoster and Tropheryma whipplei CSF PCR. CSF angiotensin converting enzyme was within normal limits and demyelination profile was negative with no oligoclonal bands detected. CSF cytology was negative for malignant cells. After admitting to recreational nitrous oxide use, the patient was evaluated for vitamin B12 deficiency. Serum vitamin B12 level was low at 105 (193-986 pg/mL) with a significantly elevated methylmalonic acid of 31000 (87-318 nmol/L) and homocysteine of 38.6 (3.2-10.7 umol/L). Intrinsic factor antibody was positive, but anti-parietal cell antibody was negative. MRI of the spine showed focal central cord T2 signal hyperintensity at C4-C5 (Figure 1). Therapy was initiated with cyanocobalamin 1000 mcg IV daily, which was later transitioned to oral cyanocobalamin 1000 mcg daily. The patient noted improvement in her symptoms following initiation of treatment.

## Discussion

Nitrous oxide use not only induces a euphoric state on its own, but can also enhance the effects of other illicit drugs [3]. Its effects last only minutes with prompt return to normal function [2-3]. These properties likely contribute to an escalating recreational use. Per the 2019 Global Drug Survey, nitrous oxide was among the top 10 drugs used over the previous 12 months, with the exclusion of alcohol and tobacco [4]. Increasing prevalence of recreational nitrous oxide use has been accompanied by growing recognition of its adverse effects including neurologic symptoms, which are reported by up to 4% of users [5].

Neurologic symptoms associated with nitrous oxide use have been attributed to vitamin B12 deficiency. Through oxidation, nitrous oxide inactivates vitamin B12. In its inactive form, vitamin B12 is unable to function as a co-factor for methionine synthase and methylmalonyl coA mutase. The former converts homocysteine into methionine, which is necessary for the production of myelin proteins, while also converting 5-methyl-tetrahydrofolate into tetrahydrofolate, which is necessary for DNA synthesis. The latter converts methyl-malonyl CoA into succinyl CoA [1]. The effect of nitrous oxide on vitamin B12 levels has been demonstrated in studies. One such study conducted by Hakimoglu and colleagues, demonstrated lower postoperative serum vitamin B12 levels in 81 patients who received anesthesia with nitrous oxide, compared to preoperative levels [6]. 

The inability to produce myelin proteins and stabilize myelin sheaths due to vitamin B12 deficiency ultimately leads to demyelination involving the peripheral and the central nervous systems [2]. Complications associated with vitamin B12 deficiency, including agranulocytosis, bone marrow suppression, psychosis, subacute combined degeneration of the spinal cord, myeloneuropathy, polyneuropathy, and peripheral neuropathy have been reported with nitrous oxide use [1-3]. Nitrous oxide is also postulated to cause damage to the neurologic system via direct mechanisms, including oxidative stress and neurotoxicity via antagonism of N-methyl-D-aspartate (NMDA) receptors [1]. 

Physical manifestations of this phenomenon include numbness, weakness, and paresthesia [1]. In a review of 18 cases of nitrous oxide toxicity, Massey and colleagues reported paresthesia and gait abnormalities as the most common neurologic presentations [7]. Findings from a survey conducted by Winstock and Ferris completed by over 16,000 recreational nitrous oxide users suggest that neurologic symptoms may be dose dependent with a 3.5% increase in reports of paresthesia with every 10% increase in nitrous oxide dose used [3]. Further evidence of this dose-dependent relationship was demonstrated by the study conducted by Hakimoglu and colleagues, which revealed higher postoperative homocysteine levels in patients who received anesthesia with nitrous oxide for greater than three hours versus those who received anesthesia for under three hours [6]. 

Patients may present with low vitamin B12 levels, as well as elevated homocysteine and methylmalonic acid levels. Classic MRI findings associated with nitrous oxide-induced vitamin B12 deficiency include T2 hyperintensity in the posterior columns, usually over several vertebral segments, with or without involvement of the lateral corticospinal tracts. These findings represent myelin sheath degeneration [2]. Treatment consists of vitamin B12/cobalamin supplementation, with response monitored both clinically (symptomatically) and via biochemical markers such as vitamin B12, methylmalonic acid, and homocysteine levels. 

Unfortunately, there is no screening test for nitrous oxide use, as its short half-life and rapid elimination make it difficult to detect [1]. As such, it is vital to obtain a thorough history regarding recreational drug use in all patients and to inquire about nitrous oxide use in those presenting with neurologic complaints. It is also important to screen those with an underlying susceptibility to vitamin B12 deficiency for nitrous oxide use, as the clinical manifestations of vitamin B12 deficiency have been noted not only in long-term nitrous oxide users, but also after a single exposure in those with susceptibility to vitamin B12 deficiency [2]. This population includes those with decreased vitamin B12 absorption, such as in those with small bowel resection, irritable bowel disease, and/or pernicious anemia, and those with reduced intake, such as in vegans and vegetarians [8]. With the growing popularity of recreational nitrous oxide use, it is imperative that proper screening for its use is implemented and prompt treatment is initiated, as response to treatment has been associated with the duration and severity of symptoms prior to initiation of treatment [3].

## Conclusions

Nitrous oxide use can cause vitamin B12 deficiency, resulting in neurologic manifestations such as myeloneuropathy and subacute combined degeneration of the spinal cord. Hence, a vitamin B12 deficiency identified in a patient presenting with neurologic complaints should prompt healthcare providers to question the patient regarding recreational drug use. Cessation of nitrous oxide use, along with vitamin B12 supplementation, can result in improvement and, in some cases, complete resolution of symptoms. 
